# Are We Ready to Implement Circadian Hygiene Interventions and Programs?

**DOI:** 10.3390/ijerph192416772

**Published:** 2022-12-14

**Authors:** Claudia R. C. Moreno, Rose Raad, Waléria D. P. Gusmão, Cristina S. Luz, Victor M. Silva, Renilda M. Prestes, Susy P. Saraiva, Lucia C. Lemos, Suleima P. Vasconcelos, Patrícia X. S. A. Nehme, Fernando M. Louzada, Elaine C. Marqueze

**Affiliations:** 1 Department of Health, Life Cycles and Society, School of Public Health, University of São Paulo, São Paulo 01246-904, Brazil; 2 Department of Psychology, Stockholm University, 106 91 Stockholm, Sweden; 3 Department of Technology of Architecture, School of Architecture and Urbanism, University of São Paulo, São Paulo 05508-080, Brazil; 4 Public Health Graduate Program, Federal University of Acre, Rio Branco 69920-900, Brazil; 5 Department of Physiology, Federal University of Paraná, Curitiba 81531-980, Brazil; 6 Department of Epidemiology, Public Health Graduate Program, Catholic University of Santos, Santos 11015-001, Brazil

**Keywords:** circadian hygiene, light–dark cycles, health lifestyle

## Abstract

Circadian hygiene, a concept not to be confused with the notion of public or social hygiene, should be discussed among experts and society. Light–dark cycles and other possible synchronizers of the human circadian timing system affect ways of life, including sleeping, eating, working and physical activity. Some of these behaviors have also been investigated individually as synchronizers (e.g., eating times). Therefore, the knowledge held today about circadian rhythms, and their implications for health, allows future perspectives in this field to be mapped. The present article summarizes the latest knowledge on factors influencing circadian rhythms to discuss a perspective for the future of health promotion based on circadian hygiene. However, it is important to highlight that circadian hygiene is the product of an imbrication of individual and societal involvement. First, it is important to adopt practices and devise public health policies in line with circadian hygiene. Second, individual healthy habits require internal rhythms to be examined. Last, the research agenda on circadian hygiene can be developed on a public as well as individual level, raising the question as to how much society is willing to embrace this change.

## 1. Introduction

Sleep hygiene is a well-established concept in sleep medicine. The same cannot be said, however, for the concept of circadian hygiene, despite advances in circadian research. The concept of circadian hygiene is seldom used, and when mentioned, closely resembles sleep hygiene. However, it is important to make a distinction between these two concepts.

Recently, an intervention study using the concept of circadian hygiene [1] with two different strategies for students to adapt their sleep habits was conducted. Although the strategies did not consider changing temporal cues from the environment, the results suggested the intervention was effective due to the lockdown during the COVID-19 pandemic. According to the authors, the lockdown reduced pressure from the “social clock”. These findings are consistent with the definition of circadian hygiene proposed in the present review, i.e., a concept in which the endogenous circadian timing system is synchronized with the environment, and external clues are included. The discussion is preceded by a brief recap of the hygiene concept. The external factors that can influence circadian rhythms are then presented.

The first mentions of the concept of sleep hygiene date back to the late 19th century with the publication of Marie de Manaceine *Sleep: Its Physiology, Hygiene and Psychology* [2]. Popularization of the concept, however, only came about in the late 70s with the book *Current Concepts: The Sleep Disorders* by Peter Hauri. The foundation of sleep hygiene comprises a list of recommendations for individual habits aimed at promoting adequate and healthy sleep [3]. In this respect, sleep specialists refer to the concept of hygiene according to its Greek origin, i.e., a set of actions taken to improve health with a view to longevity. This view is unrelated to the criticism targeting the hygienic movement, which claims that such interventions are imposed by governments, equating to eugenics or the medicalization of life [4].

The book *Circadian Rhythms: Health and Disease* published in 2015 has a captivating chapter called ‘Can we Fix a Broken Clock?’ by Schroeder and Colwell [5]. The authors discuss whether there is treatment to fix a “broken clock”. In fact, the authors’ discussion centers on how to treat sleep and circadian disorders by using light therapy, for instance. In our view, there is a need to delve deeper, given that the adoption of circadian hygiene measures does not depend on the individual alone, as is the case of sleep hygiene (not exercising close to bedtime, etc.). In other words, society as a whole needs to participate in health promotion through circadian hygiene. Thus, factors including light exposure, work hours, eating habits, physical activity and medication use affect the body according to individual traits/states, such as genome, age, gender, ethnicity and chronotype (Table 1). Circadian hygiene strategies have been shown to be successful in studies that simulate real-life conditions [6]. The temporal flexibility of society would allow such strategies to be adopted more easily. This article presents the knowledge regarding factors influencing circadian rhythms to discuss the concept of circadian hygiene as a perspective for the future of health promotion.

## 2. Examining External Factors

### 2.1. Timing of Light

Light, whether daylight/sunlight or electrical, reaching the eye, not only produces a visual response, but also activates retinal ganglion cells that are intrinsically photosensitive (ipRGCs) [7,8]. These cells are highly sensitive to short wavelengths of the visible spectrum and activate the non-visual responses to light. The non-visual responses encompass the effects of light that can influence heart rate, alertness, melatonin levels, brain activity and reset circadian rhythms [9].

The potential impact of sunlight on circadian rhythms is greater than that of electrical light since the proportion of short wavelengths in the sunlight spectrum is greater [10]. The effects arising from environmental lighting have been associated with enhanced cognitive performance, alertness, improved quality and quantity of sleep and mood [11]. The maintenance of rhythmic synchronization depends on variation in the intensity and spectrum of environmental light, making dawn, dusk and nighttime darkness equally vital for physical and mental well-being [12]. However, the potential for positive impact from sunlight has been increasingly less exploited, as humans spend most of their day within buildings, either to engage in daily activities or to seek weather protection [13]. It is noteworthy that the provision of daytime indoor lighting is not constant. 

Indoor light characteristics, such as spectrum and intensity, are influenced by external factors such as location, orientation and architectural treatment given to facades and openings, as well as by interaction with the properties of constituent materials of the surfaces of interior objects [12].

Sunlight is highly dynamic and its characteristics, such as intensity, spectrum, duration, and direction of radiation vary throughout the day and for time of the year, according to geographic position and seasonal conditions (clouds, humidity, particles suspended in atmosphere) [12,14]. Even today, with the consolidated use of LED lighting sources and their wide array of possibilities for technical and aesthetic manipulation, electric light is unable to faithfully reproduce all of the characteristics of highly variable sunlight, nor can it replicate its health benefits under artificial conditions [12]. Therefore, during the day, individuals spending most of the time indoors are exposed to overall lower levels of lighting [15]. Conversely, at night, indoor lighting levels are high relative to the desirable darkness [16]. Exposure to light at night affects the circadian timing system and, consequently, the coordination of various physiological and behavioral processes [17].

Therefore, regarding habits in urban areas, it is strongly recommended to increase time spent on outdoor activities, especially during the morning hours, when sunlight has a high concentration of short wavelengths. Longer incidence of high levels of sunlight on the retina (or bright light) during the day can also help minimize the suppression of melatonin production by electric light at night [18], by decreasing the sensitivity of intrinsically photosensitive retinal ganglion cells (ipRGCs) when stimulated by light at night. This confirms that circadian synchronization by light exposure varies, among other factors, according to photic history [19].

When indoors, seating arrangements placed as close to windows as possible should be prioritized. To promote adequate stimulus of retinal cells, even when in indoor spaces, increased daytime brightness is recommended. This can be achieved by primarily exploiting daylight and, when needed, complementing this with electric lighting enriched in the blue light wavelength, especially early in the morning [20]. Throughout the day, ideally the levels of light and short wavelengths in the lighting composition should be reduced, with lowest exposure at night, especially in the final hours before bedtime [21].

Finally, it is important to note that the usual metrics for calculating photopic lighting are no longer appropriate for quantifying the non-visual effects of light. To address these concerns, a number of calculation tools have been developed, including the measures circadian light (Cla) and circadian stimulus (CS), equivalent melanopic lux (EML) and melanopic equivalent daylight illuminance (melanopic EDI models). These metrics represent a major advance to standardize the design and implementation of human-healthy lighting systems. For example, EML, devised by the Lucas group [22], and melanopic EDI, recommended by the International Commission on Illumination—CIE [23], are based on the spectrum of action of melanopsin.

### 2.2. Eating Times

Sleeping times and waking hours, as well as mealtimes properly coordinated with the environmental light–dark cycle, are essential for maintaining health [24]. A network that includes homeostasis, sleep–wakefulness, feeding and fasting is under the control of the circadian timing system [24] and finely coordinates the temporal regulation of metabolism. Thus, the timing of food intake (chrononutrition) has emerged as a possible key to maintaining metabolic health and goes far beyond caloric intake. Daily rhythms are involved in nutrient assimilation via specific genes and have circadian rhythmicity of expression, at times maximizing the use of nutrients while at others eliminating unusable materials as metabolic waste [25].

The recent literature raises two important points with significant supporting evidence [26]. Firstly, circadian misalignment between the body’s internal and external environment affects food metabolism, such as digestion and absorption of food, as well as energy metabolism. Metabolic alterations induced by circadian misalignment contribute to weight gain, obesity and the development of the metabolic syndrome, among other health problems. A second point concerns the effect of programmed feeding, that is, time-restricted feeding (TRF), initially studied in animal models [27]. The protocols used provide restricted feeding at specific times in order to produce an anticipatory response from the animals in search of food. This protocol, known as food anticipatory activity (FAA) led to a phase advance in gene expression, especially in peripheral oscillators such as the organs involved in food digestion and absorption processes.

Restricting eating times (or eating window) can act as a protective factor against obesity and metabolic disorders. This phenomenon occurs regardless of meal size and composition, and the strategy is attracting increasing attention as a tool for preventing obesity [28] and chronic disease [29] in general.

Methods to characterize meal times in the context of the internal circadian time have been developed. Zerón-Rugerio et al. [28] used a measure of the interval of time between dinner and the midpoint of sleep (midsleep). Interestingly, an interval of 6 h between dinner and midsleep was associated with the lowest values of adiposity. The authors concluded that dining 6 h before midsleep seems to help avoid obesity, depending on circadian and nutritional factors. A study of night-shift workers found that for every hour less between the last meal and sleep onset, there was a 24 min increase in diurnal sleep duration [30].

Time-restricted feeding has also been suggested as an effective strategy for glycemic control and weight loss, with positive results for insulin resistance and improvements in beta-cell function among men with prediabetes, and in 24 h glucose levels among people with obesity [31]. In addition, a weight-loss study concluded that the eating interval might also affect metabolism [32].

Therefore, many factors can influence metabolic health. It is crucial to elucidate not only the effect of the caloric content of meals but also their timing, given this can determine the physiological response to nutrient availability [25]. Time-restricted feeding may be considered as an option for intermittent fasting (IF), where there is alternation between periods of feeding and prolonged fasting. IF has been suggested as an alternative to reduce weight and regulate appetite and insulin levels. Irregular feeding times can cause disruption of the circadian system and adverse health effects [33,34].

### 2.3. Timing of Physical Activity

Research on the effectiveness of physical exercise for enhancing quality of life is widely discussed in the literature [35]. A recent study of older people with sleep disorders found an association between walking and better sleep quality in women [36]. The indication of physical exercises for the older population to promote successful biological and, particularly, cognitive aging, seems to be more effective when optimal duration and times are determined.

Not only has the older population benefited from regular exercise. Weight reduction, modulation of neurotransmitters [37], neuroprotection [38], lowering of nocturnal blood pressure [39] and increased psychological well-being are recognized factors for improving sleep quality and duration in different genders and ages [40]. Although it is well documented that regular physical exercise is beneficial for several health indicators, timing of exercise should be investigated. Studies involving exposure to nocturnal physical exercise under constant conditions resulted in significant phase delays of circadian hormonal rhythms [41]. These results were corroborated by a recent study aimed to establish phase-response curves related to the timing of exercise, with phase delays from 19:00 to 22:00 h [42]. In addition, the PRC showed phase advances at 07:00 h and from 13:00 to 16:00 h, whereas and minimal shifts around 16:00 and 02:00 h [42].

Body temperature, for example, is a variable that can influence physical exercise performance [43] and exhibits circadian temporal variation, generally reaching higher levels in the late afternoon [44]. This variation can be tracked using parameters of core temperature and skeletal muscle temperature [45]. Thermoregulatory responses seem to have a greater capacity to dissipate body heat in the afternoon than in the morning [46], which could explain the greater performance in physical exercises in the afternoon compared to morning [47,48,49]. This information does not mean that everyone should only exercise in the afternoon, regardless of performance. Considering well-being and quality of life from the perspective of circadian phenotypes (especially morning types), light and moderate exercises can also be performed in the morning if this is pleasant and better suited to individual demands. However, according to the PRC study [42] mentioned earlier it is important to choose times with minimal shifts. Therefore, the application of circadian hygiene in the physical exercise routine can promote better results in all individuals, from patients to high-performance athletes.

### 2.4. Chronobiotics

Chronopharmacology therapy aims to adapt the time of administration of drugs or active substances to the user’s endogenous circadian timing to improve treatment, as well as reduce the risk of adverse effects or toxicity associated with the use of medications. Although chronopharmacology may be considered part of circadian hygiene, optimizing drugs according to the time of day is, in fact, circadian medicine. In this field, modified-release drugs use technologies that aim to potentiate the peaks of drug action at specifically determined times of the day [50]. Studies have shown that several classes of drugs have their actions altered by endogenous rhythmicity, and there are drugs that can shift the biological rhythms of individuals. This group includes medications that act on the cardiovascular system as long-acting medications with an angiotensin receptor blocker to control bedtime hypertension [51], for example. In addition, drugs for glycemic control, such as empagliflozin, have metabolism slightly higher at evening [52] as well as drugs for cancer treatment, such as oral fluoropyrimidines, which are better tolerated with systemic drug exposure at night [53]. Another example are drugs for inflammatory or autoimmune diseases, such as prednisone, prednisolone and non-steroidal anti-inflammatory drugs, having the best potential when administrated at bedtime [54,55].

However, the relevant issue regarding circadian hygiene in chronopharmacology is chronobiotics, used as a strategy to synchronize endogenous rhythms to environmental cycles. Chronobiotics can be hormones or drugs that act as zeitgebers, changing the circadian phase of endogenous rhythms. Exogenous melatonin and melatonin receptor agonists can act as chronobiotics, shifting central circadian time and peripheral oscillators [56]. Melatonin, with its biological functions of adjusting biological rhythmicity, is the most widely accepted chronobiotic used in medicine [57].

There is evidence that exogenous melatonin has the ability to reduce sleep latency and sleep fragmentation; however, reports of increased sleep duration or sleep consolidation are still controversial [58]. The effects of exogenous melatonin are associated with low reports of tolerance, dependence or feeling of a “hangover” the next day, which makes its use a positive resource, if necessary [59].

### 2.5. Social Times

In addition to external factors that shift clock timing, commented above, there are social habits (work, school) that affect our perceived light–dark cycle. Thus, social habits indirectly shift clock timing since they affect the sleep–awake times. In this section, we will focus on school and work times.

As mentioned earlier, the sleep–wake cycle is influenced by the light–dark cycle, as well as by external factors such as temperature, noise and social factors [18,19]. Thus, incompatibility between adolescent sleep patterns and morning school schedules negatively impacts academic performance [60,61]. This occurs because adolescents experience a sleep-phase delay compared to other stages of life according to the normal process of ontogenesis, which alters the sleep–wake cycle throughout the lifespan.

Similarly, there is a mismatch between work hours and the diurnal pattern of the circadian timing system when individuals work night shifts. Nevertheless, in a globalized economy demanding 24 h customer service, the impact of shift work is increasingly relevant. However, historically, production costs and the availability of human resources have been given priority in work schedules, with little consideration for the effects on workers’ health [62]. This prioritization is problematic since shift work is known to be associated with sleep restriction and circadian rhythm misalignment. The repercussions on the health of shift workers have been reported in thousands of studies, representing a risk factor for cardiovascular and metabolic problems and cancer [63]. However, it is still unclear whether the negative health effects of shift work are mediated by circadian misalignment, sleep restriction and social misalignment, or all of these factors [63].

According to a systematic review, the number of years of shift work seems to be significantly associated with a greater chance of workers having mental health problems [64]. However, most of the studies reviewed failed to provide information on the number of nights participants worked each year. This information is important to estimate the dose–response relationship for any disease related to shiftwork, a point made abundantly clear in the ensuing discussion among members in the last monograph about night shift work as an etiologic agent for cancer [65].

Protective measures against health risks for shift workers involving interventions proved heterogeneous [66]. Careful preparation of work schedules that take into account the characteristics of workers can help mitigate risk, safeguard sleep opportunities, align work schedules with biological rhythm, and allow adequate recovery time after each shift [67]. The fatigue risk management system, for instance, seeks to mitigate fatigue by ensuring sleep, providing a suitable place for napping, organizing shifts to ensure restorative sleep, collecting data on sleep, as well as detecting symptoms of fatigue [66]. Naps can be used to reduce sleep pressure and help maintain alertness and performance during work shifts. This is a strategy that can be employed both before and during shifts. However, it is essential to make sure time for napping incorporates a period for recovery from sleep inertia (15–30 min) [68].

Many measures have been suggested to minimize the undesirable effects of light on circadian rhythms at night outlined earlier. Recommendations include the use of orange light glasses on the commute home, digital screen filters that block blue light, and change of lighting in the work environment [69]. In addition, some studies have investigated the administration of exogenous melatonin as a strategy to shift the circadian system and if appropriately timed can help adjust to shift work schedules [70,71]. Recently, a study of night workers found that exogenous melatonin decreased circadian misalignment and reduced body weight among early types, without any change in the participants’ calorie intake or physical activity levels [72]. Regular physical activity also seems to reduce fatigue of night shift workers [68]. Moreover, healthy food intake and adequate meal times for the individual´s circadian rhythm and working hours may help shift workers [68].

In the case of adolescents and school hours, there are also individual and social types of interventions that could be implemented, such as sleep education and changes to school start times [73,74,75]. Moreover, the prolonged use of electronic devices with illuminated screens in the evening are associated with a reduction in nighttime sleepiness, poorer sleep quality, delayed melatonin production, increased body temperature, decreases in nocturnal melatonin production and daytime attention levels [76,77]. This is a common practice among adolescents and young adults, and it should be avoided.

## 3. Discussion

Drawing on the knowledge held today about circadian clocks and how their timing can be shifted, and the implications for health when clocks are misaligned in relation to environmental cycles, future perspectives of this field can be mapped. The concept of circadian hygiene can be useful for implementing interventions and programs in different settings. Broader than the concept of sleep hygiene, circadian hygiene-based interventions and programs would have to take into account necessary changes in workplaces, schools, hospitals, as well as public spaces such as parks and sports arenas.

Evidence shows that food intake goes beyond the caloric content of meals. Eating at night can lead to increased risk of developing diseases. However, the complexity of the topic reveals the need for future studies considering the chrononutrition context. Moreover, social changes and support are needed to adopt strategies on food-intake timing. For example, increasing the time between the last meal and onset of sleep will only be possible if people have the opportunity to eat earlier in the evening. This aspect is directly related to work shift duration and commuting time. Long working hours, in conjunction with inefficient transport systems, may preclude the ideal interval between last meal and sleep onset.

Working hours are key to circadian hygiene. Contemporary society needs to rethink the need for non-essential sectors working 24/7. Supermarkets, gyms and shops in general do not need to work around the clock. Clearly, a number of key sectors, such as safety, health care, and energy supplies, cannot simply stop working. In this case, some recommendations can be adopted to minimize the negative effects of working at night. A recent consensus paper summarized the existing literature and recommended that night shift schedules should be limited to ≤3 consecutive night shifts, shift intervals should be ≥11 h, and shift duration ≤9 h [78]. These recommendations are aimed at reducing the risk of injuries and the possibly of breast cancer. The authors also suggested that pregnant women should work no more than one night shift per week [76]. In short, circadian hygiene, as related to shiftwork, could be divided into individual and organizational measures.

The same logic might be applied to eating and exercising. Several elements should be taken into account before defining “the best time” to exercise. For example, respecting individual temporal characteristics, such as individual daytime preference and alertness level, at times scheduled for physical activities, can ensure better circadian hygiene [79]. As is the case for nutritional concepts, circadian hygiene in physical exercise will only be possible with social changes. This involves, for example, the provision of safe and pleasant public places to practice physical activities and flexible working hours that respect individual preferences.

Chronobiotics should also take into account the variability of intra- and inter-individual biological rhythms, as factors determining dose adjustment. Advances in chronopharmacology will enable more rational and precise use of chronobiotics, considering the time of day for administration.

## 4. Future Directions

In order to implement interventions and programs related to circadian hygiene, it is important to propose practices and public health policies that incorporate this knowledge. Public spaces for outdoor leisure and the encouragement of this pursuit are both essential (Table 1). This does not mean, however, that indoor public spaces should be forgotten. Workplaces, schools and even indoor spaces for leisure, must be adequately illuminated in order to increase the entry of sunlight and reduce the impact of electric light on circadian rhythms at night. Furthermore, shift work should not be adopted in sectors where there is no justifiable need for it.

It is important to recognize that worker performance cannot be the same when working at three o’clock in the afternoon as at three o’clock in the morning. The food provided in company canteens should also consider this knowledge. In addition, the pharmaceutical industry should invest in developing drugs to optimize their effects according to the timing of administration (and genes involved).

A second point is individual awareness that internal rhythms should be respected as a healthy habit:

- Avoiding meals close to bedtime and having breakfast are measures that should be encouraged.
- Exercising according to individual preferences for activity and rest is healthy, while lack of exercise is not.
- Keeping in mind that sleep needs and circadian rhythms phase change with age help people maintain healthy habits.
- Considering individual differences should be a premise.
- A morning type should ideally work in the morning and not at night.
- The prolonged use in the evening of electronic devices with illuminated screens should be avoided.

The future hygiene research agenda can be mapped (Table 2). Once again, collective/public and individual levels can be distinguished. Little or no progress has been made in studies on public lighting, which should take into account not only safety aspects, but also its effects on the circadian rhythms of residents living close to street lighting. Hospitals represent a paradox, while healthcare workers treat patients, hospital managers appear unconcerned with the circadian rhythms of staff and patients. Research in hospitals to promote the health of staff/patients via a healthy environment is necessary to adapt these settings in line with the latest knowledge [80].

## 5. Conclusions

In summary, circadian hygiene involves educating not only individuals, but also society as a whole, encompassing intersectional, economic and social approaches. What we need to know is whether society is ready to embrace changes in policies promoting environments and organizations that respect our internal timing.

## Figures and Tables

**Table 1:** Circadian hygiene factors/behaviors and their characteristics.

Factors/Behaviors	Characteristics	Individual Aspects
Timing of light	Location (indoors/outdoors); time; wavelengths (short/medium/long); intensity; type (LED/incandescent/fluorescent); illuminance	Age, photic history, chronotype, race, genome, and sex.
Eating times	Time; intervals; proximity to bedtime; content; quantity	
Timing of physical activity	Time; duration; frequency; type of exercise	
Chronobiotics	Dose–response; timing; desired response	
Social times	Start/end times; shift intervals; shift duration; number of consecutive nights (night-shift work)	

**Table 2:** Research agenda. To refine recommendations and put these into practice on a large scale, further knowledge must be acquired on the effects of several factors on the circadian system, taking into account age, sex, ethnic and chronotype differences.

Timing of light	Identify appropriate time of exposure, duration, light intensity, wavelength, type of light source (LED, incandescent or fluorescent) and illuminance to maintain entrainment. This includes indoor and outdoor lighting (streets; houses; hospitals; workplaces, etc.).
Eating times	Identify appropriate time, intervals, content, quantity, and proximity to bedtime of meals to avoid undesirable metabolic effects. Knowledge of an individual’s internal timing is crucial to define their ideal eating window or timing of meals.
Timing of physical activity	Identify appropriate time, duration, frequency and type of exercise to promote better synchronization of circadian rhythmicity.
Chronobiotics	Identify dose–response and ideal timing to obtain the desirable response of an effective and safe chronobiotic for each individual.
Social times	Identify the “dose–response” of negative outcomes, i.e., number of work nights associated with cancer among night-shift workers; to determine the appropriate time to go to school. Knowledge of an individual’s internal timing is crucial to define sleep/awake times.

## Data Availability

Not applicable.
